# Letter to the Editor Re: Comino, I., *et al*. Nutrients 2013, *5*, 4250–4268

**DOI:** 10.3390/nu5124964

**Published:** 2013-12-05

**Authors:** Kamil K. Hozyasz

**Affiliations:** Pediatric Department, Institute of Mother and Child, 17a Kasprzaka Str., Warsaw, Poland; E-Mail: khozyasz@verco.com.pl

Dear Editor,

I read with interest the recently published review article titled “The gluten-free diet: testing alternative cereals tolerated by celiac patients” by Comino *et al.* [1] in Nutrients. However, there is very sparse data on so-called minor cereals and no data on candidate wild graminoids, which have been gathered in the past.

Availability of palatable gluten-free grain foods is expected to grow in coming years and this will provide many opportunities for agriculture companies to market new cereals that are tasty and affordable [2]. Three major trends in the gluten-free cereal market have been observed in the past 20 years: 1. introduction of new cultivars of formerly known gluten-free species (e.g., naked *Avena sativa*); 2. introduction of species of minor cereals from Africa and Asia (e.g., teff, proso millet, white fonio (*Digitaria exilis*), black fonio (*Digitaria iburua*), finger millet, jungle rice, Kodo millet, adlay, cattail millet); 3. introduction of species which, as wild graminoids, were previously gathered (e.g., Indian ricegrass, also known as montina—*Achnatherum hymenoides*) [3].

Indian ricegrass was a widely used food plant of Indian tribes in the USA and nowadays gluten-free all-purpose baking flour is marketed from this cultivated cereal. The Middle European traditions of culinary use of wild graminoids (manna grass—*Glyceria fluitans*, plicate sweet-grass—*Glyceria nocata*, cheat—*Bromus secalinus*, tribe *Bromeae*, and green bristle grass—*Setaria glauca*, tribe *Paniceae*) form an area for future research which may provide valuable gluten-free cereals. *Glyceria* is one of the main genera in the small, isolated tribe *Meliceae* [4]. In Middle Europe from medieval times until at least the 18th century, *Glyceria* seeds, the most expensive cereal, constituted an important part of taxes paid by peasants to landowners and was even exported [5,6]. Manna grass was used to make gruel (boiled with milk), desserts with butter and bread, which were highly valued. The importance of manna grass products as a Polish cuisine speciality was reported by foreigners visiting the country in the past [5]. Based on taxonomy, manna grass can be considered gluten-free; however, further studies are needed to measure its safety and usefulness for celiac patients. It needs to be stressed that it can take years to select cultivars of *Glyceria fluitans* suitable for intensive agriculture.
